# Association Between Atopic Dermatitis and Colorectal Cancer Risk: A Korean Population-Based Study

**DOI:** 10.3390/biomedicines13102538

**Published:** 2025-10-17

**Authors:** Ho Suk Kang, Kyeong Min Han, Joo-Hee Kim, Ji Hee Kim, Hyo Geun Choi, Dae Myoung Yoo, Ha Young Park, Nan Young Kim, Mi Jung Kwon

**Affiliations:** 1 Division of Gastroenterology, Department of Internal Medicine, Hallym University Sacred Heart Hospital, Hallym University College of Medicine, Anyang 14068, Republic of Korea; hskang76@hallym.or.kr; 2 Hallym Data Science Laboratory, Hallym University College of Medicine, Anyang 14068, Republic of Korea; km.han@hallym.ac.kr (K.M.H.); ydm@hallym.ac.kr (D.M.Y.); 3 Division of Pulmonary, Allergy, and Critical Care Medicine, Department of Internal Medicine, Hallym University Sacred Heart Hospital, Hallym University College of Medicine, Anyang 14068, Republic of Korea; luxjhee@hallym.or.kr; 4 Department of Neurosurgery, Hallym University Sacred Heart Hospital, Hallym University College of Medicine, Anyang 14068, Republic of Korea; kimjihee.ns@gmail.com; 5 Suseo Seoul E.N.T. Clinic, 10, Bamgogae-ro 1-gil, Gangnam-gu, Seoul 06349, Republic of Korea; mdanalytics@naver.com; 6 Department of Pathology, Busan Paik Hospital, Inje University College of Medicine, Busan 47392, Republic of Korea; hy08.park@gmail.com; 7 Hallym Institute of Translational Genomics and Bioinformatics, Hallym University Medical Center, Anyang 14068, Republic of Korea; honeyny@hallym.or.kr; 8 Department of Pathology, Hallym University Sacred Heart Hospital, Hallym University College of Medicine, Anyang 14068, Republic of Korea

**Keywords:** colorectal cancer, atopic dermatitis, nested case–control study, bigdata, risk factor

## Abstract

**Background/Objectives:** Atopic dermatitis (AD) is a common chronic inflammatory skin disease that may influence cancer risk through immune dysregulation and chronic inflammation. The association between AD and colorectal cancer (CRC) remains unclear, with previous studies reporting conflicting results. Evidence from East Asian populations, where CRC incidence is rapidly rising, is particularly limited. **Methods:** We conducted a nested case–control study using the Korean National Health Insurance Service–National Sample Cohort (2002–2019). A total of 9920 incident CRC cases were identified and matched 1:4 with 39,680 controls by age, sex, income, and residential region. AD was defined using diagnostic codes and prescription records. Overlap propensity score weighting was applied to minimize confounding, and weighted logistic regression was used to estimate odds ratios (ORs) with 95% confidence intervals (CIs). **Results:** AD was not significantly associated with CRC risk (adjusted OR = 0.97, 95% CI: 0.91–1.04). The null association was consistent across subgroups stratified by age, sex, comorbidity burden, and allergic comorbidities. Sensitivity analyses yielded similar results. **Conclusions:** In this large, nationwide, population-based study, AD did not exhibit a significant connection with the risk of CRC. This null association remained consistent across multiple subgroups and sensitivity analyses, suggesting that AD may not play a substantial role in colorectal carcinogenesis. However, the observational design and lack of detailed lifestyle information may limit causal interpretation.

## 1. Introduction

Colorectal cancer (CRC) stands as the third leading cancer diagnosis worldwide and the second primary cause of cancer mortality [1]. In Korea, CRC remains a critical health problem, with 28,111 new cases (11.0% of all cancers) and 8774 deaths reported in 2018, corresponding to an age-standardized incidence rate of 33.7 per 100,000, exceeding the global average [2,3]. Despite advances in screening and prevention, CRC incidence in East Asia continues to rise and CRC remains a major public health challenge in East Asia [4], highlighting the importance of identifying modifiable risk and protective factors.

Atopic dermatitis (AD) is a long-standing inflammatory condition of the skin, impacting up to 10% of adults and 20% of children [5] and the disease is especially prevalent in East Asia and has been associated with various socioeconomic burdens, including sleep disturbance, psychological stress, reduced quality of life, and decreased work productivity [6,7]. Increasing evidence suggests that AD is not merely a cutaneous condition but a systemic disorder associated with autoimmune, psychiatric, metabolic, and possibly malignant comorbidities [5,8,9,10]. The potential link between AD and cancer is biologically plausible, but the direction of association remains uncertain [11]. On one hand, immune surveillance theory proposes that heightened immune responsiveness in allergic conditions may reduce cancer risk by facilitating early elimination of transformed cells [12]. On the other hand, chronic inflammation theory posits that persistent immune activation may promote carcinogenesis through DNA damage, oxidative stress, and dysregulated cytokine signaling [13,14].

Beyond epidemiological associations, molecular pathways may provide biological plausibility for a link between AD and CRC. AD is characterized by a type 2 inflammatory milieu, driven largely by Th2 cells, eosinophils, mast cells, and dendritic cells. These cells secrete cytokines such as interleukin (IL)-4, IL-5, IL-13, IL-31, and thymic stromal lymphopoietin (TSLP) [15], which disrupt epithelial barrier function, promote chronic inflammation, and influence downstream pathways including NF-κB, JAK/STAT, and MAPK signaling [16]. In the intestinal microenvironment, chronic type 2 immune activation may interact with colonic epithelial cells and resident immune populations, altering tumor immune surveillance and fostering a pro-tumorigenic niche [17]. Conversely, eosinophil- and macrophage-mediated anti-tumor immunity has also been observed in preclinical models of colon cancer, suggesting that the net effect of AD-related immune activity on CRC risk may be context-dependent [18]. These molecular insights support the need for population-based studies to clarify whether the systemic immune dysregulation in AD translates into measurable CRC risk.

Epidemiological evidence has been conflicting across populations and study designs. A Taiwanese nationwide cohort (2000–2013) reported that AD patients had a 26% higher CRC risk (hazard ratio 1.26, 95% CI 1.14–1.40), with consistent effects across age and sex strata [19]. In contrast, a meta-analysis pooling over half a million individuals found that allergic conditions overall showed a 12% reduction in CRC risk (relative risk 0.88, 95% CI 0.83–0.92) and mortality [20]. Likewise, the U.S. Multiethnic Cohort study (over 215,000 participants, 4834 incident CRC cases) reported a 14% lower CRC incidence (relative risk 0.86, 95% CI 0.80–0.92) and 20% lower CRC mortality, with protective associations in whites, African Americans, Native Hawaiians, and Japanese Americans, but not in Latinos, suggesting ethnic-specific variability [21].

In addition, evidence from European multicenter cohorts reinforced the potential protective role of allergic disease [22]. In a pooled analysis of over 400,000 adults from several European countries, individuals with a history of allergic disease (including AD, allergic rhinitis, or asthma) demonstrated a modestly reduced CRC risk (HR = 0.90), though the effect size varied by country and was attenuated after adjusting for smoking, body mass index, and diet [22]. This indicates that environmental exposures, lifestyle, and healthcare systems may modulate the allergy–cancer relationship across populations.

Moreover, emerging genomic evidence strengthens the plausibility of an AD–CRC link. A recent Mendelian randomization and transcriptomic study identified *TET2* as a shared gene signature, showing that genetically predicted AD was elated to a 17% lower likelihood of CRC (odds ratio (OR) 0.83, 95% CI 0.75–0.94) [23]. Downregulation and hypermethylation of TET2 were further linked to worse CRC prognosis, while high TET2 expression enriched immune-regulatory pathways (Th1/Th2/Th17 differentiation, antigen presentation, NF-κB signaling) [23].

Given the conflicting results across Asian and Western studies, the uncertain contribution of ethnicity, and the limited evidence from Korean populations, we conducted a nationwide nested case–control study using the NHIS–National Sample Cohort to clarify the association between AD and CRC in Korea.

## 2. Materials and Methods

### 2.1. Ethical Approval, Data Acquisition, and Study Framework

The research protocol received approval from the Institutional Review Board of Hallym University Sacred Heart Hospital (IRB No. 2022-10-008), and the obligation to obtain written informed consent was waived in light of the use of de-identified data. All procedures of the study were performed in line with established ethical principles and guidelines. To examine the link between prior history and exposure status in determining outcomes, a nested case–control design was judged to be suitable [24].

For this retrospective nested case–control study, we used the Korean National Health Insurance Service–National Sample Cohort (KNHIS-NSC), a representative cohort comprising nearly 2.2% of the Korean population, with follow-up spanning 2002 to 2019 [25]. Using random sampling, the cohort was established and stratified into 1476 strata based on age (18 groups), sex, and income level (41 groups), thereby guaranteeing national representativeness. Subjects were tracked until 2019, with censoring applied for death or emigration. The KNHIS-NSC provides an extensive and stable resource compiled from national insurance claims and governmental health databases.

### 2.2. Outcome: Colorectal Cancer

CRC cases were defined as individuals with ICD-10 codes C18–C20 or D010–D012 and a special claim code for serious cancer (V193 or V194) recorded between 2005 and 2019. Under the Korean national insurance scheme, V-codes are issued only to patients with officially verified cancer who are registered for critical illness coverage. This dual-coding strategy, widely implemented in Korean administrative data analyses, increases diagnostic validity and case detection by minimizing the inclusion of suspected or provisional cancer cases [26].

### 2.3. Exposure: Atopic Dermatitis

AD participants were identified if they had a diagnosis of AD (ICD-10 code L20) accompanied by records of prescribed AD-related medications (e.g., topical or systemic corticosteroids, calcineurin inhibitors, or antihistamines). This dual requirement, widely used in Korean claims-based research, was adopted to enhance diagnostic specificity and reduce potential misclassification.

### 2.4. Participant Selection and Matching

**Figure 1:**
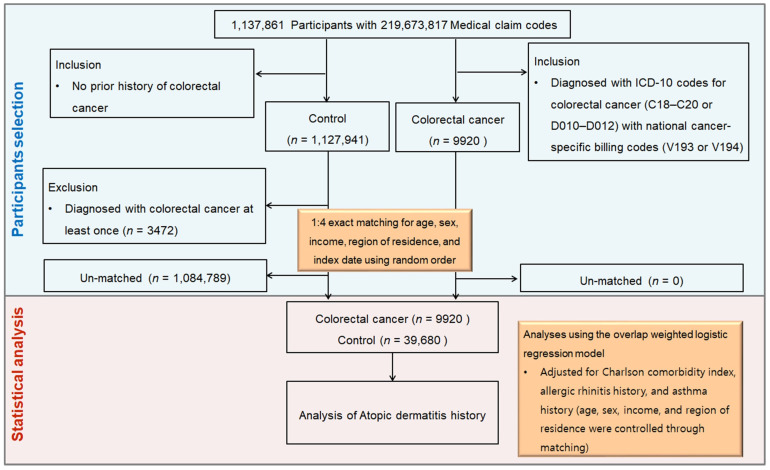
Schematic illustration of the participant selection process in the present study. Among a total of 1,137,861 participants, 9920 CRC participants were matched with 39,680 control participants for age, sex, income, and region of residence.

From 1,137,861 individuals and 219,673,817 medical claims, we identified 9920 CRC patients between 2002 and 2019. The control cohort comprised individuals free of CRC (n = 1,127,941). Each individual with CRC was paired with four controls according to age, sex, income, and place of residence (1:4 ratio). Random selection minimized bias, and the index date of CRC diagnosis was assigned to matched controls for temporal alignment. For every CRC case, the index date corresponded to the date on which both CRC-specific ICD-10 codes (C18–C20 or D010–D012) and the cancer-related special claim codes (V193 or V194) were simultaneously assigned in the claims database. Controls were given the index date of the CRC cases to which they were matched. Accordingly, every matched patient–control set shared an identical index date. Subsequent to this matching step, propensity scores were estimated from all baseline covariates, with overlap weighting applied to further mitigate residual confounding and optimize covariate balance. Ultimately, 39,680 controls and 9920 CRC patients were included in the final analysis (Figure 1).

### 2.5. Covariates

Age was categorized into 18 five-year groups, and income into five levels (1 = lowest to 5 = highest). Residential areas were classified into 16 administrative regions and dichotomized as urban or rural. Comorbidity burden was assessed using the Charlson Comorbidity Index (CCI, range 0–29), excluding all malignancies to avoid overlap with the cancer outcome of interest [27]. Allergic comorbidities—including allergic rhinitis (ICD-10: J30.1–J30.4) and asthma (ICD-10: J45–J46)—were also identified.

### 2.6. Statistical Analyses

To minimize confounding and improve comparability between CRC cases and controls, we applied overlap weighting based on propensity scores. Propensity scores were estimated using multivariable logistic regression models including all baseline covariates. Overlap weights were assigned as 1–propensity score for CRC cases and as propensity score for controls, thereby emphasizing individuals with similar probabilities of exposure [28]. Covariate balance after weighting was assessed using standardized mean differences, with values < 0.1 indicating adequate balance [29]. The association between AD and CRC was examined using logistic regression models applied to the matched dataset with overlap weights. Both crude and adjusted ORs with 95% confidence intervals (CIs) were estimated. Adjusted models additionally included unmatched covariates—CCI score, allergic rhinitis, and asthma—to account for residual confounding. Subgroup analyses stratified by key covariates were conducted to assess potential effect modification. All analyses were performed using SAS version 9.4 (SAS Institute, Cary, NC, USA), and statistical significance was defined as a two-tailed *p* < 0.05.

## 3. Results

### 3.1. Baseline Characteristics

As shown in Table 1, the baseline characteristics of 9920 individuals with CRC and 39,680 matched controls were compared after 1:4 matching on age, sex, income level, and region of residence. Post-matching, all covariates reached a standardized difference of 0.00, demonstrating strong comparability between groups. Overlap weighting further eliminated any residual imbalance, with standardized differences for all covariates reduced to zero. Key characteristics such as the CCI, allergic rhinitis, and asthma were equally distributed. Collectively, these results indicate that our matching and weighting strategies were highly effective in reducing confounding and ensuring the robustness of the analyses.

### 3.2. Relationship Between Colorectal Cancer and Atopic Dermatitis

As shown in Table 2, we conducted a propensity score overlap-weighted multivariable logistic regression analysis to assess the association between CRC and AD. The crude OR for CRC among individuals with AD was 0.95 (95% CI, 0.88–1.03; *p* = 0.214), and the adjusted OR was 0.97 (95% CI, 0.91–1.04; *p* = 0.387). These findings indicate that there was no statistically significant association between AD and the risk of CRC.

### 3.3. Subgroup Analyses

**Figure 2:**
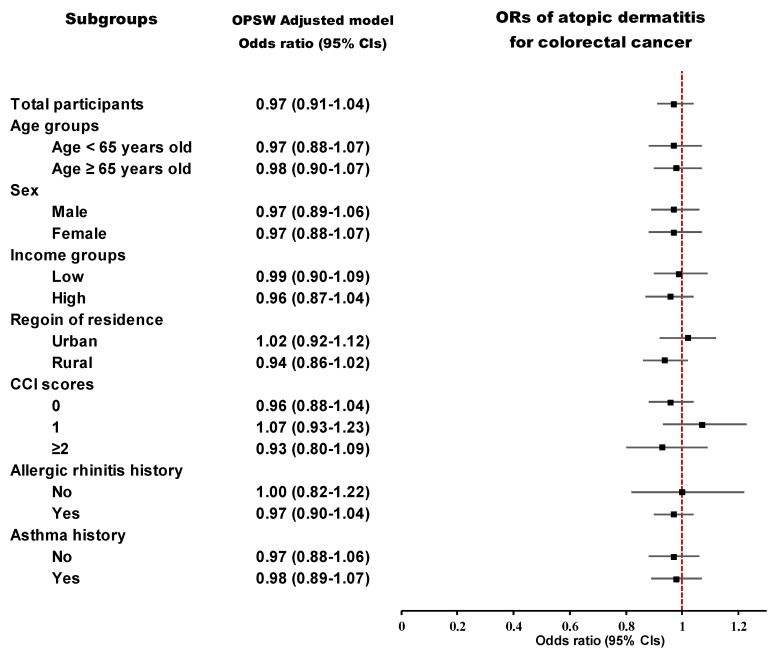
Forest plot of odds ratios (95% confidence intervals) for the association between atopic dermatitis and colorectal cancer, including subgroup analyses by age, sex, income, residential area, Charlson comorbidity index, allergic rhinitis history, and asthma history.

Subgroup analyses revealed no statistically significant associations between atopic dermatitis and colorectal cancer across all demographic and clinical strata (Table 2; Figure 2). The adjusted odds ratios remained close to 1.0 in every subgroup, indicating a consistent lack of association. For example, the adjusted OR was 0.98 (95% CI, 0.90–1.07; *p* = 0.609) for participants aged ≥65 years, and 0.97 (95% CI, 0.88–1.07; *p* = 0.502) for females. Similar null associations were observed in males (OR, 0.97; 95% CI, 0.89–1.06; *p* = 0.561), low-income groups (OR, 0.99; 95% CI, 0.90–1.09; *p* = 0.859), and rural residents (OR, 0.94; 95% CI, 0.86–1.02; *p* = 0.138). Across all strata defined by CCI scores, allergic rhinitis, and asthma history, no subgroup demonstrated a statistically meaningful deviation from the null.

These findings suggest that atopic dermatitis is not independently associated with colorectal cancer risk, regardless of demographic, socioeconomic, or clinical characteristics.

## 4. Discussion

In this large, nationally representative Korean cohort, we observed no significant association between AD and CRC (adjusted OR = 0.97, 95% CI 0.91–1.04). The null result was consistent across all examined subgroups—including age, sex, socioeconomic status, comorbidity burden, and coexisting allergic diseases—and was robust in sensitivity analyses applying alternative AD definitions. These findings suggest that AD is unlikely to be an independent determinant of CRC risk in the Korean population.

Previous studies have focused on the potential association between allergic diseases and cancer. Some studies have reported that allergic conditions may serve as protective factors against cancer development by enhancing immune surveillance [11,12,30]. In contrast, other studies have suggested that allergies could increase the risk of cancer [31,32]. While the prevailing hypothesis posits that heightened immune responses associated with allergies may aid in recognizing and eliminating abnormal cells, allergies can also reflect a chronic immune dysregulation that may promote carcinogenesis under certain conditions [33]. The relationship between allergic diseases and CRC specifically has therefore remained inconclusive, and the role of AD in CRC development is still debated [22,34].

Ethnic background may help explain these inconsistencies. In Western populations, allergic conditions have often been associated with reduced CRC risk [12,21,22,32]. For example, the U.S. Multiethnic Cohort reported a 14% reduction in CRC incidence and 20% lower mortality among individuals with allergic diseases, though effects varied by race/ethnicity, with null associations in Latinos [21]. Similarly, a European multicenter case–control study involving over 1900 CRC cases and 4000 controls found a reduced CRC risk in allergic individuals, particularly for rectal cancer (colon cancer OR = 0.88, 95% CI 0.67–1.14; rectal cancer OR = 0.64, 95% CI 0.44–0.92) [22]. A prospective meta-analysis further consolidated these findings, reporting a pooled relative risk of 0.88 for CRC incidence and mortality among individuals with allergic diseases [32]. Importantly, however, these protective associations may not be attributable to AD specifically. Recent Mendelian randomization studies linking AD to reduced CRC risk via *TET2*-mediated immune and epigenetic regulation were based largely on Western datasets, and their generalizability to East Asian populations remains uncertain [23].

In Asian populations, however, results have been more heterogeneous. Taiwanese nationwide cohorts have reported both increased and decreased CRC risks among AD patients, likely reflecting methodological and contextual differences such as diagnostic coding, statistical modeling, and healthcare utilization [10,19]. Notably, one Taiwanese study observed a 26% increased CRC risk in AD patients [19], based on ICD-9 coding and a Fine–Gray competing-risk model that adjusted for lifestyle proxies and colonoscopy frequency, yet residual confounding by healthcare utilization and detection bias could not be ruled out. Our study provides balanced evidence on the AD–CRC association amid conflicting findings [10,12,19,21,22,32]. Key strengths include the large sample size, comprehensive insurance data, and reduced selection bias through 1:4 matching, with additional validity from overlap-weighted logistic regression, which minimized bias, achieved excellent covariate balance [28], and strengthened confidence in the null association.

Genetic factors may also contribute to population-specific findings in the AD–CRC association. Distinct AD susceptibility loci have been reported in East Asians compared with Europeans, including *FLG*, *IL13*, and *DOCK8*, which regulate epithelial barrier integrity and type 2 immune responses [35,36]. Such differences could influence how AD-related inflammation interacts with colorectal carcinogenesis [35,36]. Epigenetic regulation adds further complexity: downregulation or hypermethylation of *TET2* has been associated with worse CRC prognosis [23], whereas genetically predicted AD has been linked to reduced CRC risk through *TET2*-mediated immune modulation [37]. Importantly, reduced *TET2* expression in CRC correlates with diminished cytotoxic T-cell infiltration and altered macrophage polarization, fostering a pro-tumorigenic microenvironment [38], while higher *TET2* expression enhances antigen presentation, Th1/Th2/Th17 differentiation, and NF-κB signaling, thereby strengthening anti-tumor immunity [38]. Thus, variations in allele frequencies, immune profiles, and epigenetic regulation across populations may explain why protective associations are more frequently observed in Western cohorts, whereas null or increased risks are reported in Asian studies.

Nonetheless, several limitations must be acknowledged. First, AD diagnoses were based on insurance claims data, which may be subject to misclassification or underreporting. To improve case ascertainment, we required both diagnostic codes and prescription records; however, claims data do not capture important clinical details such as disease severity, physician-confirmed diagnostic criteria, or laboratory markers. Consequently, some degree of non-differential misclassification is likely. Because such misclassification would occur similarly among CRC cases and controls, it may have attenuated true associations and biased the ORs toward the null, which should be considered when interpreting our findings. Second, the absence of validated severity measures for AD limited our ability to examine potential dose–response effects. Although proxies such as dermatology consultation frequency, systemic therapy use (e.g., cyclosporine, methotrexate, biologics), or referral to tertiary care could serve as indicators of severity, these data were not consistently available in our dataset. Future studies integrating clinical registries or severity proxies will be essential to determine whether more severe AD differentially influences CRC development. Third, the study did not include information on AD severity, which may be an important modifier of cancer risk [39]. In addition, because this was a retrospective observational study, causal inference is inherently limited. Although we applied robust statistical approaches—such as 1:4 propensity score matching and overlap weighting—to address confounding and selection bias, unmeasured confounding and uncertainties in temporal relationships cannot be completely ruled out. Fourth, both CRC and AD are known to be strongly influenced by dietary factors such as processed meats and red meat consumption [40,41,42]; however, our analysis did not account for lifestyle and environmental factors such as smoking, alcohol consumption, diet, or physical activity, which are well-established determinants of CRC risk [43]. The omission of these confounders may have biased our findings. For instance, greater exposure to smoking or alcohol among AD patients could lead to an overestimation of CRC risk, while protective behaviors such as healthier diets or higher physical activity could attenuate risk estimates. Because these opposing influences may counterbalance one another, the net direction of bias remains uncertain. Nevertheless, the absence of such data underscores the possibility of residual confounding and highlights the importance of future studies integrating lifestyle and environmental information. Fifth, our findings may be limited in generalizability, as the study population consisted solely of individuals of Korean ethnicity. Another important point to consider is the stage of CRC. Unfortunately, information on CRC staging (I–IV) was not available in the Korean National Health Insurance Service–National Sample Cohort, which relies on administrative claims data. Thus, our study could not stratify patients according to tumor stage. This is a limitation, as AD-related immune dysregulation may theoretically exert different effects depending on tumor progression. For example, stronger immune surveillance in early stages might suppress tumor initiation, whereas chronic inflammation could contribute to progression in later stages. To date, however, there is very limited literature directly linking AD with CRC stage-specific risk, and no large-scale population-based analyses have established such an association. Future studies integrating clinical registries with detailed tumor characteristics and immune profiling will be essential to clarify whether AD differentially affects CRC initiation versus progression.

## 5. Conclusions

In conclusion, this nationwide nested case–control study found no significant association between AD and CRC risk in the Korean population. Our findings suggest that AD alone does not substantially influence CRC risk and, based on current evidence, changes to existing CRC screening recommendations are not warranted. Nevertheless, several take-home messages emerge. First, the relationship between AD and CRC is complex and may be modified by chronic inflammation, immune dysregulation, genetic/epigenetic susceptibility, and ethnic background. Second, residual confounding from unmeasured lifestyle and environmental factors and the absence of AD severity and CRC stage data represent important limitations. Finally, divergent results across Taiwanese, Korean, European, and U.S. populations, together with emerging genomic evidence, highlight the need for population-specific investigations. Future research integrating clinical severity measures, lifestyle variables, genomic data, and cancer staging will be essential to determine whether certain subgroups of AD patients may require tailored CRC prevention or surveillance strategies.

## Figures and Tables

**Table 1:** General Characteristics of Participants.

Characteristics		Before PS Overlap Weighting Adjustment			After PS Overlap Weighting Adjustment		
		Colorectal Cancer	Control	Standardized Difference	Colorectal Cancer	Control	Standardized Difference
Age (n, %)				0.00			0.00
	0–4	1 (0.01)	4 (0.01)		1 (0.01)	1 (0.01)	
	5–9	N/A	N/A		N/A	N/A	
	10–14	3 (0.03)	12 (0.03)		2 (0.03)	2 (0.03)	
	15–19	1 (0.01)	4 (0.01)		1 (0.01)	1 (0.01)	
	20–24	8 (0.08)	32 (0.08)		6 (0.08)	6 (0.08)	
	25–29	26 (0.26)	104 (0.26)		21 (0.26)	21 (0.26)	
	30–34	94 (0.95)	376 (0.95)		75 (0.95)	75 (0.95)	
	35–39	180 (1.81)	720 (1.81)		144 (1.81)	144 (1.81)	
	40–44	359 (3.62)	1436 (3.62)		287 (3.62)	287 (3.62)	
	45–49	578 (5.83)	2312 (5.83)		461 (5.82)	461 (5.82)	
	50–54	968 (9.76)	3872 (9.76)		773 (9.76)	773 (9.76)	
	55–59	1242 (12.52)	4968 (12.52)		991 (12.51)	991 (12.51)	
	60–64	1393 (14.04)	5572 (14.04)		1111 (14.02)	1111 (14.02)	
	65–69	1488 (15.00)	5952 (15.00)		1187 (14.98)	1187 (14.98)	
	70–74	1471 (14.83)	5884 (14.83)		1175 (14.84)	1175 (14.84)	
	75–79	1060 (10.69)	4240 (10.69)		847 (10.69)	847 (10.69)	
	80–84	672 (6.77)	2688 (6.77)		539 (6.80)	539 (6.80)	
	85+	376 (3.79)	1504 (3.79)		301 (3.80)	301 (3.80)	
Sex (n, %)				0.00			0.00
	Male	5933 (59.81)	23,732 (59.81)		4736 (59.80)	4736 (59.80)	
	Female	3987 (40.19)	15,948 (40.19)		3184 (40.20)	3184 (40.20)	
Income (n, %)				0.00			0.00
	1 (lowest)	1990 (20.06)	7960 (20.06)		1589 (20.06)	1589 (20.06)	
	2	1253 (12.63)	5012 (12.63)		1000 (12.62)	1000 (12.62)	
	3	1562 (15.75)	6248 (15.75)		1246 (15.74)	1247 (15.74)	
	4	2059 (20.76)	8236 (20.76)		1644 (20.76)	1644 (20.76)	
	5 (highest)	3056 (30.81)	12,224 (30.81)		2441 (30.82)	2441 (30.82)	
Region of residence (n, %)				0.00			0.00
	Urban	4447 (44.83)	17,788 (44.83)		3550 (44.82)	3550 (44.82)	
	Rural	5473 (55.17)	21,892 (55.17)		4370 (55.18)	4370 (55.18)	
CCI score (Mean, SD)		3.60 (2.44)	0.99 (1.65)	0.09	3.58 (2.18)	1.07 (0.77)	0.00
Allergic rhinitis history (n, %)		7722 (77.84)	31,987 (80.61)	0.07	6212 (78.43)	6212 (78.43)	0.00
Asthma history (n, %)		3951 (39.83)	16,362 (41.23)	0.03	3176 (40.11)	3176 (40.11)	0.00
Atopic dermatitis (n, %)		783 (7.89)	3284 (8.28)	0.01	628 (7.93)	645 (8.14)	0.01

Abbreviations: CCI, Charlson Comorbidity Index; PS, propensity score; N/A, not applicable; SD, standard deviation.

**Table 2:** Crude and overlap propensity score weighted odds ratios of atopic dermatitis for colorectal cancer.

Characteristics		N of Colorectal Cancer	N of Control	Odd Ratios for Colorectal Cancer (95% Confidence Interval)			
		(Exposure/Total, %)	(Exposure/Total, %)	Crude	*p*	Overlap Weighted Model †	*p*
Total participants (*n* = 49,600)							
	AD	783/9920 (7.9)	3284/39,680 (8.3)	0.95 (0.88–1.03)	0.214	0.97 (0.91–1.04)	0.387
	Control	9137/9920 (92.1)	36,396/39,680 (91.7)	1		1	
Age < 65 years old (*n* = 24,265)							
	AD	338/4853 (7.0)	1425/19,412 (7.3)	0.94 (0.84–1.07)	0.367	0.97 (0.88–1.07)	0.543
	Control	4515/4853 (93.0)	17,987/19,412 (92.7)	1		1	
Age ≥ 65 years old (*n* = 25,335)							
	AD	445/5067 (8.8)	1859/20,268 (9.2)	0.95 (0.86–1.06)	0.394	0.98 (0.90–1.07)	0.609
	Control	4622/5067 (91.2)	18,409/20,268 (90.8)	1		1	
Male (*n* = 29,665)							
	AD	441/5933 (7.4)	1838/23,732 (7.7)	0.96 (0.86–1.07)	0.425	0.97 (0.89–1.06)	0.561
	Control	5492/5933 (92.6)	21,894/23,732 (92.3)	1		1	
Female (*n* = 19,935)							
	AD	342/3987 (8.6)	1446/15,948 (9.1)	0.94 (0.83–1.06)	0.334	0.97 (0.88–1.07)	0.502
	Control	3645/3987 (91.4)	14,502/15,948 (90.9)	1		1	
Low income group (*n* = 24,025)							
	AD	361/4805 (7.5)	1485/19,220 (7.7)	0.97 (0.86–1.09)	0.622	0.99 (0.90–1.09)	0.859
	Control	4444/4805 (92.5)	17,735/19,220 (92.3)	1		1	
High income group (*n* = 25,575)							
	AD	422/5115 (8.3)	1799/20,460 (8.8)	0.93 (0.83–1.04)	0.218	0.96 (0.87–1.04)	0.313
	Control	4693/5115 (91.7)	18,661/20,460 (91.2)	1		1	
Urban resident (*n* = 22,235)							
	AD	370/4447 (8.3)	1493/17,788 (8.4)	0.99 (0.88–1.12)	0.875	1.02 (0.92–1.12)	0.756
	Control	4077/4447 (91.7)	16,295/17,788 (91.6)	1		1	
Rural resident (*n* = 27,365)							
	AD	413/5473 (7.5)	1791/21,892 (8.2)	0.92 (0.82–1.02)	0.123	0.94 (0.86–1.02)	0.138
	Control	5060/5473 (92.5)	20,101/21,892 (91.8)	1		1	
CCI scores = 0 (*n* = 30,415)							
	AD	424/5448 (7.8)	2051/24,967 (8.2)	0.94 (0.85–1.05)	0.291	0.96 (0.88–1.04)	0.31
	Control	5024/5448 (92.2)	22,916/24,967 (91.8)	1		1	
CCI scores = 1 (*n* = 10,617)							
	AD	216/2600 (8.3)	664/8017 (8.3)	1.00 (0.85–1.18)	0.968	1.07 (0.93–1.23)	0.361
	Control	2384/2600 (91.7)	7353/8017 (91.7)	1		1	
CCI scores ≥ 2 (*n* = 8568)							
	AD	143/1872 (7.6)	569/6696 (8.5)	0.89 (0.74–1.08)	0.234	0.93 (0.80–1.09)	0.382
	Control	1729/1872 (92.4)	6127/6696 (91.5)	1		1	
Non-allergic rhinitis history (*n* = 9891)							
	AD	92/2198 (4.2)	315/7693 (4.1)	1.02 (0.81–1.30)	0.849	1.00 (0.82–1.22)	0.968
	Control	2106/2198 (95.8)	7378/7693 (95.9)	1		1	
Allergic rhinitis history (*n* = 39,709)							
	AD	691/7722 (8.9)	2969/31,987 (9.3)	0.96 (0.88–1.05)	0.368	0.97 (0.90–1.04)	0.339
	Control	7031/7722 (91.1)	29,018/31,987 (90.7)	1		1	
Non-asthma history (*n* = 29,287)							
	AD	396/5969 (6.6)	1624/23,318 (7.0)	0.95 (0.85–1.06)	0.369	0.97 (0.88–1.06)	0.473
	Control	5573/5969 (93.4)	21,694/23,318 (93.0)	1		1	
Asthma history (*n* = 20,313)							
	AD	387/3951 (9.8)	1660/16,362 (10.1)	0.96 (0.86–1.08)	0.515	0.98 (0.89–1.07)	0.59
	Control	3564/3951 (90.2)	14,702/16,362 (89.9)	1		1	

Abbreviations: AD, atopic dermatitis; CCI, Charlson Comorbidity Index. † Adjusted for age, sex, income, region of residence, CCI score, allergic rhinitis history, and asthma history.

## Data Availability

Restrictions apply to the availability of these data. The data were obtained from the Korean National Health Insurance Sharing Service (NHISS) and are available at https://nhiss.nhis.or.kr (accessed on 9 September 2025).
